# Preexisting Humoral Immunity Cross-Reacting with SARS-CoV-2 Might Prevent Death Due to COVID-19 in Critical Patients

**DOI:** 10.3390/jcm11133870

**Published:** 2022-07-04

**Authors:** Taro Yamashita, Tetsuro Shimakami, Kouki Nio, Takeshi Terashima, Masaki Okajima, Takumi Taniguchi, Takashi Wada, Masao Honda, Toshifumi Gabata, Kenji Ota, Katsunori Yanagihara, Shuichi Kaneko

**Affiliations:** 1 Department of General Medicine, Kanazawa University Hospital, Kanazawa 920-8641, Japan; 2 Center for Education in Community Medicine, Kanazawa University Hospital, Kanazawa 920-8641, Japan; shimakami@m-kanazawa.jp; 3 Department of Gastroenterology, Kanazawa University Hospital, Kanazawa 920-8641, Japan; nio@m-kanazawa.jp (K.N.); tera@m-kanazawa.jp (T.T.); mhonda@m-kanazawa.jp (M.H.); skaneko@m-kanazawa.jp (S.K.); 4 Department of Emergency and Disaster Medicine, Kanazawa University Hospital, Kanazawa 920-8641, Japan; mmokaji@gmail.com; 5 Department of Anesthesiology and Intensive Care Medicine, Kanazawa University Hospital, Kanazawa 920-8641, Japan; ttaniyan@med.kanazawa-u.ac.jp; 6 Department of Nephrology and Laboratory Medicine, Kanazawa University Hospital, Kanazawa 920-8641, Japan; twada@m-kanazawa.jp; 7 Department of Radiology, Kanazawa University Hospital, Kanazawa 920-8641, Japan; gabata@med.kanazawa-u.ac.jp; 8 Department of Laboratory Medicine, Nagasaki University Hospital, Nagasaki 852-8501, Japan; kenjiotamd@nagasaki-u.ac.jp (K.O.); k-yanagi@nagasaki-u.ac.jp (K.Y.)

**Keywords:** SARS-CoV-2, COVID-19, humoral immune memory, mortality

## Abstract

The preexistence of humoral immunity, which cross-reacts with severe acute respiratory syndrome coronavirus 2 (SARS-CoV-2) protein due to prior endemic low-pathogenic human coronavirus infection, has been reported, but its role in coronavirus disease 2019 (COVID-19) outcomes remains elusive. We evaluated serum samples obtained from 368 patients before the pandemic and 1423 independent serum samples from patients during the pandemic. We found that approximately 6~13% and 1.5% of patients had IgG cross-reactivity to the SARS-CoV-2 spike and nucleocapsid proteins in both cohorts. We evaluated the IgG cross-reactivity to the SARS-CoV-2 spike and nucleocapsid proteins in 48 severe or critical COVID-19 patients to evaluate if the elevation of IgG was evoked as a primary response (IgG elevation from 10 days after antigen exposure) or boosted as a secondary response (IgG elevation immediately after antigen exposure). Approximately 50% of patients showed humoral immune responses to the nucleocapsid protein of SARS-CoV-2. Importantly, none of the critically ill patients with this humoral immunity died, whereas 40% of patients without this immunity did. Taken together, subjects had humoral immunity to SARS-CoV-2 nucleocapsid but not spike before the pandemic, which might prevent critically ill COVID-19 patients from dying.

## 1. Introduction

Coronavirus disease 2019 (COVID-19), caused by severe acute respiratory syndrome coronavirus 2 (SARS-CoV-2) infection, is a global pandemic, and it has reached almost every country worldwide [1]. Since it was first reported in late 2019 in China, about 525 million people have been infected with SARS-CoV-2 and 6.3 million people have died across world (as of 18 May 2022, according to the database provided by https://coronavirus.jhu.edu/map.html). This disease is characterized by a wide range of severe symptoms, and approximately 14 and 5 percent of patients developed severe and critical diseases, respectively [1], especially during the pandemic period driven by virulent SARS-CoV-2 alpha and Delta variants. Patients with COVID-19 exhibit various disease severity which most strongly correlates with survival, and older patients generally show more severe disease and worse clinical outcomes compared with younger patients [2]. Although severe disease rates of COVID-19 declined after the emergence of the SARS-CoV-2 Omicron variant, several risk factors remained for severe illness, including older age, male, diabetes mellitus, hypertension, and obesity. 

The mortality rate of COVID-19 in East Asia is relatively low compared with other demographic areas. For example, the number of deaths due to COVID-19 in USA and Japan were 1 million and 30 thousand, respectively (as of 18 May 2022, according to the database provided by https://coronavirus.jhu.edu/map.html). One hypothesis to explain this regional difference is the preexistence of humoral immunity cross-reacting with severe acute respiratory syndrome coronavirus 2 (SARS-CoV-2) protein due to prior endemic low-pathogenic human coronavirus infection (hCoVs) in East Asia. Consistent with this, recent evidence suggests the preexistence of humoral immunity cross-reacting with SARS-CoV-2 protein, potentially due to prior infection with endemic low-pathogenic hCoVs [3,4,5,6]. However, its role in COVID-19 outcomes remains elusive. In this study, we evaluated IgG responses to SARS-CoV-2 proteins using serum samples obtained from 368 patients before the pandemic and 1423 independent serum samples from patients during the pandemic. We further evaluated the clinical outcome of 48 COVID-19 patients with severe or critical disease in relation to IgG responses to SARS-CoV-2 proteins.

## 2. Materials and Methods

### 2.1. Patients and Samples

We utilized 368 (from 2004 to 2019) and 1423 (in 2020) serum samples obtained from patients who visited Kanazawa University Hospital without medical history of COVID-19. We further used 219 serum samples from 368 patients obtained as matched samples in 2020. In these patients, informed consent was obtained by an opt-out method. We enrolled 48 patients who were admitted to Kanazawa University Hospital or Nagasaki University Hospital with a diagnosis of COVID-19. A total of 27 and 21 patients were diagnosed with severe or critical disease on admission, respectively. Since these patients were admitted to the hospital from December 2020 to March 2021, all these patients were considered as infected with the SARS-CoV-2 alpha variant prevalent in that time in Japan. All serum samples were obtained on the day of hospitalization. The average time from symptom onset to hospitalization in this cohort was about 7 days. All COVID-19 patients provided written informed consent before enrollment. The study protocol was approved by the ethics committee of each hospital. The serum samples were stored in a −20 °C freezer and used for the quantification of IgG titers by enzyme-linked immunosorbent assay (ELISA).

### 2.2. SARS-CoV-2 IgG Antibody Quantification

We used the Quo Research ELISA system to evaluate the IgG titer against recombinant severe acute respiratory syndrome coronavirus 2 (SARS-CoV-2) nucleocapsid protein (amino acids 1–419) (IgG N) and spike protein (amino acids 16–1213) (IgG S) (Cellspect Co., Ltd., Morioka, Japan). Cutoff values of IgG N (0.5) and IgG S (0.2) at an optical density of 450 nm absorbance were determined according to the manufacturer’s recommendations.

### 2.3. SARS-CoV-2 S1 and ACE2 Binding Neutralization Assay

The neutralizing activity of serum samples on the spike receptor-binding domain (S1 RBD) and angiotensin-converting enzyme 2 (ACE2) binding was measured using the SARS-CoV-2 Neutralizing Antibodies Detection kit (Adipogen AG, Liestal, Switzerland) according to the manufacturer’s instructions. Percentage of inhibition was calculated according to the manufacturer’s instructions. The positive control serum sample from a COVID-19 patient with neutralizing activity to S1 RBD and ACE2 binding was purchased from RayBiotech Life, Inc. (Peachtree Corners, GA, USA). The negative control serum sample was obtained from a physician without a history of COVID-19. Six serum samples were obtained at 7 days after the second vaccination shot from physicians who received the BNT162b2 vaccine.

### 2.4. Multi-Alignment Analysis

The amino acid alignment of the nucleocapsid antigen (N), spike S1 domain, and spike S2 domain of SARS-CoV-2 used for ELISA was compared with the SARS-CoV-2, SARS-CoV, and alpha (229E and NL63) and beta (OC43 and HKU1) human coronaviruses. An analysis was performed using GENETYX software ver. 13.1.1 (Genetyx, Tokyo, Japan), and similarity (gray boxes) and identity (black boxes) corresponding to the N RNA-binding domain (amino acids 50–173 of SARS-CoV-2), the S1 receptor-binding domain (amino acids 319–541 of SARS-CoV-2), and the S2 domain (amino acids 707–1213 of SARS-CoV-2) were analyzed. GenBank protein accession numbers for nucleocapsid RNA-binding domain were YP_009724397 (SARS-CoV-2), AAZ67043 (SARS-CoV), P33469 (OC-43), ABD96199 (HKU1), AIW52699.1 (229E), and YP_003771 (NL63). GenBank protein accession numbers for the S1 receptor-binding domain were QIH45093 (SARS-CoV-2), ACU31032 (SARS-CoV), ARE30017 (OC-43), BBA20986 (HKU1), AWH62679 (229E), and AKT07952 (NL63). GenBank protein accession numbers for the S2 domain were QIH45093 (SARS-CoV-2), ACU31032 (SARS-CoV), ARE30017 (OC-43), BBA20986 (HKU1), AWH62679 (229E), and AKT07952 (NL63).

### 2.5. Statistical Analysis

Kaplan–Meier curves of cumulative deaths in patients diagnosed with critical COVID-19 on admission were compared using log-rank tests with GraphPad Prism software ver. 9.2.0 (GraphPad Software, San Diego, CA, USA). Two-sided *p*-values of 0.05 or less were considered statistically significant.

## 3. Results

### 3.1. Homology of Amino Acid Sequences among SARS-CoV-2, SARS-CoV, and Alpha and Beta hCoVs

**Figure 1:**
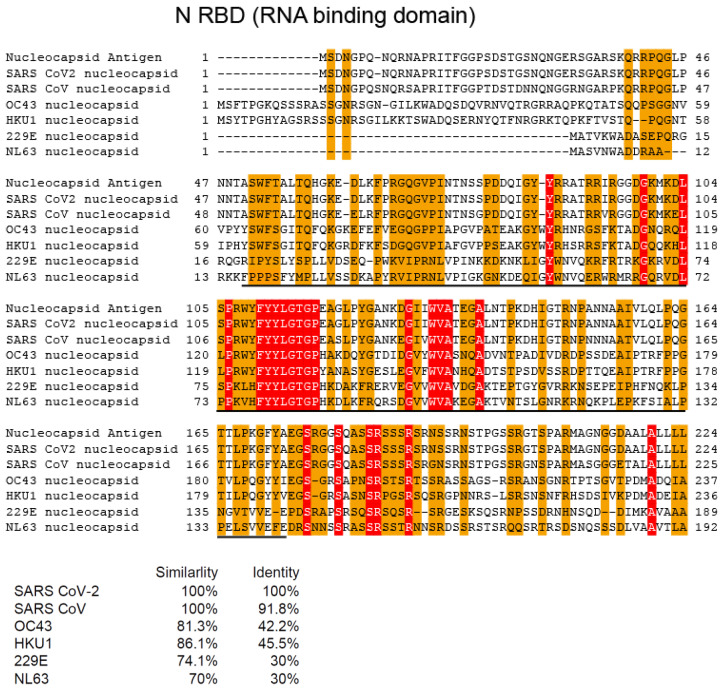
Multi-alignment analysis of the nucleocapsid RNA-binding domain of SARS-CoV-2, SARS-CoV, and alpha (229E and NL63) and beta (OC43 and HKU1) human coronaviruses. Similarity and identity are indicated as orange boxes and red boxes, respectively.

**Figure 2:**
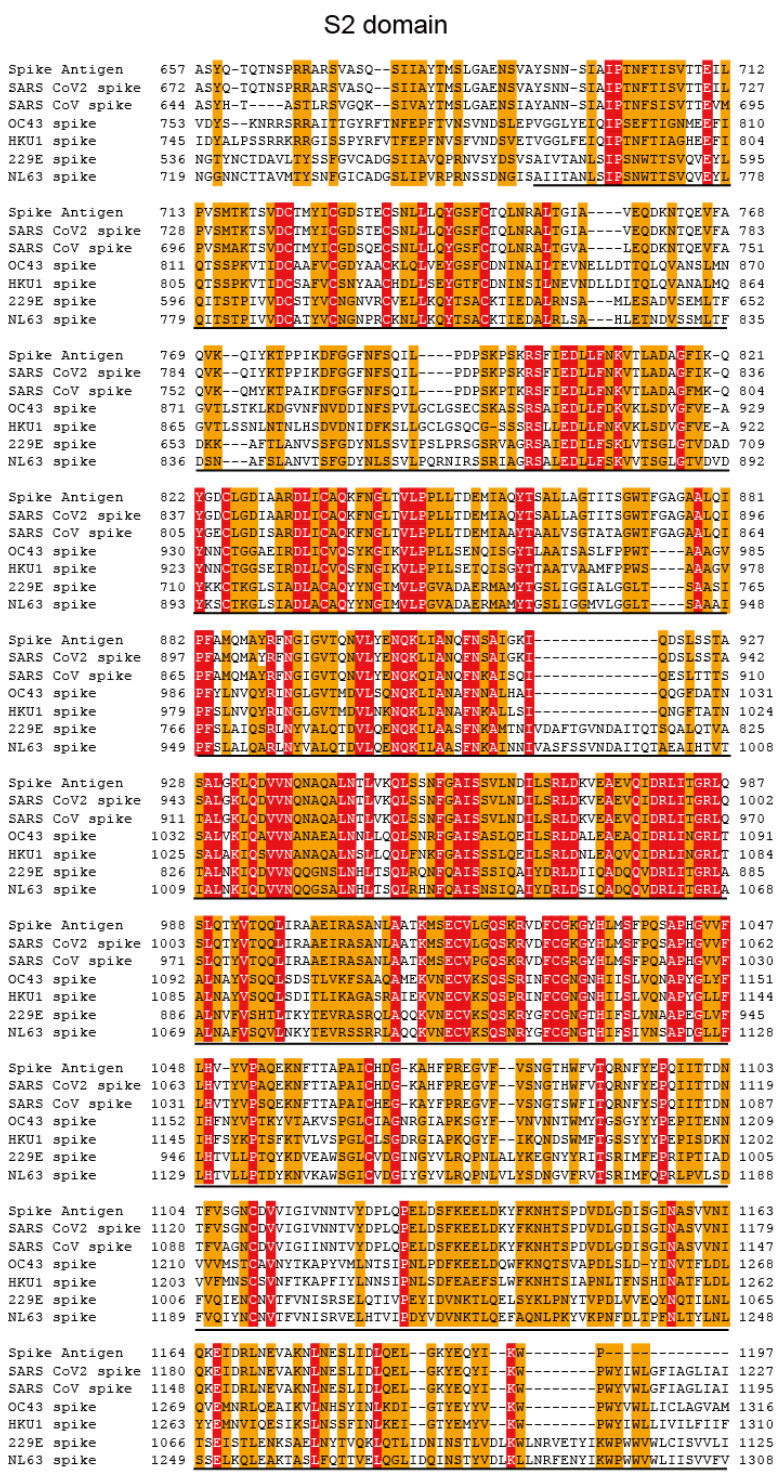

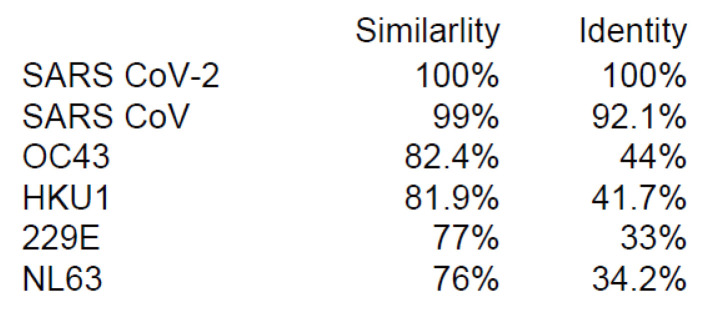
Multi-alignment analysis of the spike S2 domain of SARS-CoV-2, SARS-CoV, and alpha (229E and NL63) and beta (OC43 and HKU1) human coronaviruses. Similarity and identity are indicated as orange boxes and red boxes, respectively.

**Figure 3:**
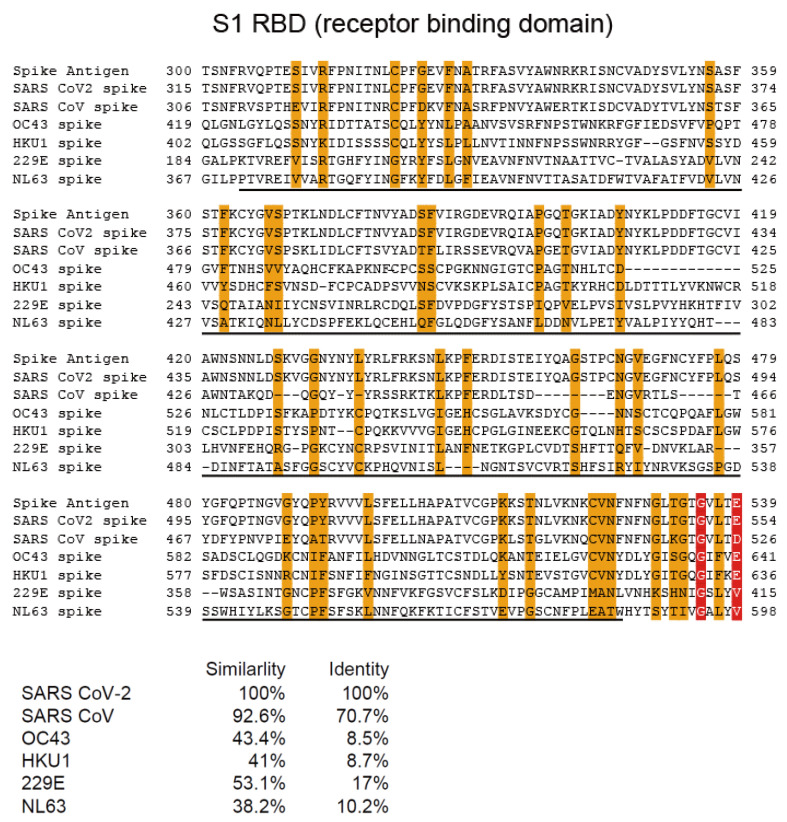
Multi-alignment analysis of the spike S1 receptor-binding domain of SARS-CoV-2, SARS-CoV, and alpha (229E and NL63) and beta (OC43 and HKU1) human coronaviruses. Similarity and identity are indicated as orange boxes and red boxes, respectively.

We performed a multi-alignment analysis of the nucleocapsid RNA-binding domain of SARS-CoV-2, SARS-CoV, and alpha (229E and NL63) and beta (OC43 and HKU1) human coronaviruses. Multi-alignment analysis revealed relatively conserved amino acid sequences among SARS-CoV-2, SARS-CoV, and alpha and beta hCoVs in the N RNA-binding domain and part of the S2 domain (Figure 1 and Figure 2). In contrast, no homology was detected in the S1 receptor-binding domain (RBD) (Figure 3).

### 3.2. Humoral Immunity to SARS-CoV-2 before and during Pandemic

**Figure 4:**
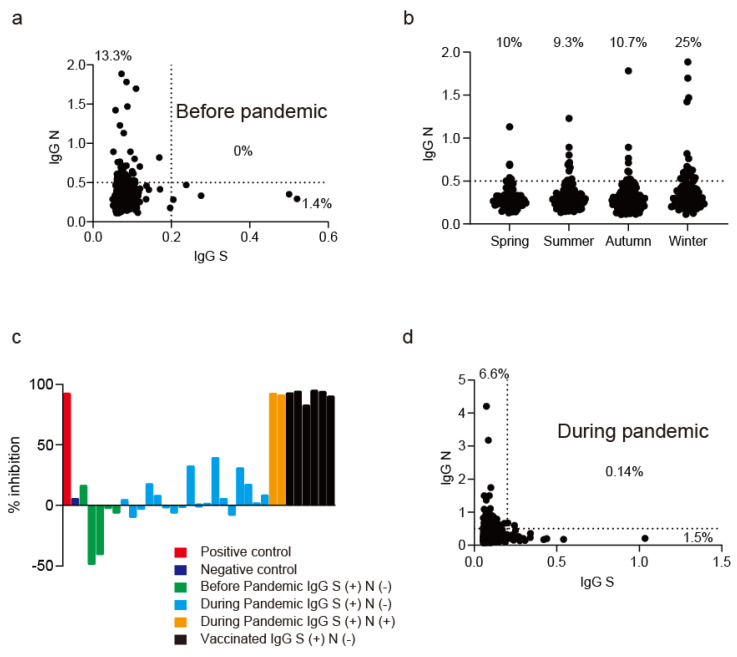
Humoral immunity to SARS-CoV-2 N and S proteins in sera obtained before and during pandemic. (**a**) IgG N and S titers of serum samples obtained before the COVID-19 pandemic (*n* = 368). X and Y axes indicate the OD values at 450 nm absorbance evaluated by the Quo Research ELISA system. (**b**) IgG N titers of serum samples obtained before the COVID-19 pandemic according to season (*n* = 368). Serum samples obtained from March to May, June to August, September to November, and December to February were regarded as samples obtained in spring, summer, autumn, and winter, respectively. (**c**) Neutralizing activity of serum samples regarded as IgG S (+) before and during the pandemic on S1 RBD and ACE2 binding. (**d**) IgG N and S titers of serum samples obtained during the COVID-19 pandemic (*n* = 1423). X and Y axes indicate the OD values at 450 nm absorbance evaluated by the Quo Research ELISA system.

Since we found the relatively conserved region of the N RNA-binding domain and a part of S2 domain among SARS-CoV-2, SARS-CoV and endemic low-pathogenic hCoVs, we decided to evaluate the presence of IgG cross-reacting to the N, S1 and S2 domains in sera obtained before the SARS-CoV-2 pandemic. Among 368 serum samples obtained before 2019, 13.3% already contained IgG bound to N (IgG N) whereas IgG bound to S (IgG S) were rarely detected (1.4%) (Figure 4a). IgG N titers varied according to season, becoming highest in winter (25%) and lowest in summer (9.3%) (Figure 4b), suggesting that the acquisition of IgG N occurred in the winter when hCoVs were seasonally prevalent. The neutralizing activity of serum samples on SARS-CoV-2 S1 RBD and ACE2 binding was measured, and IgG S-positive sera showed no inhibitory effects on binding to SARS-CoV-2 S1 RBD and ACE2 (Figure 4c, green bars). These data suggested that the IgG S detected before the pandemic most likely recognized the S2 domain conserved in SARS-CoV-2 and hCoVs (Figure 2), and this immune memory acquisition was a rare event without an inhibitory effect on virus-receptor binding. Humoral immunity to SARS-CoV-2 N and S was tested in an additional 1423 patients without a medical history of COVID-19 during the pandemic. Again, 6.6% and 1.5% of their serum samples showed positivity to IgG N and IgG S, respectively (Figure 4d). Among them, 18 of 1423 cases had IgG S but not IgG N (Figure 4c, light blue bars), and again these sera showed no inhibitory effects on binding to SARS-CoV-2 S1 RBD and ACE2. Only two patients (0.14%) had both IgG N and S, and only these serum samples showed neutralizing activity against S1 RBD and ACE2 binding (Figure 4c, orange bars). These data indicated that the presence of IgG N or S alone designates the humoral immune memory to N or S induced by endemic hCoVs, whereas simultaneous IgG responses to N and S reflect the SARS-CoV-2 post-infection status during the pandemic. Thus, the asymptomatic SARS-CoV-2 infection rate in Kanazawa Japan in November and December 2020 was estimated as 0.14%.

**Figure 5:**
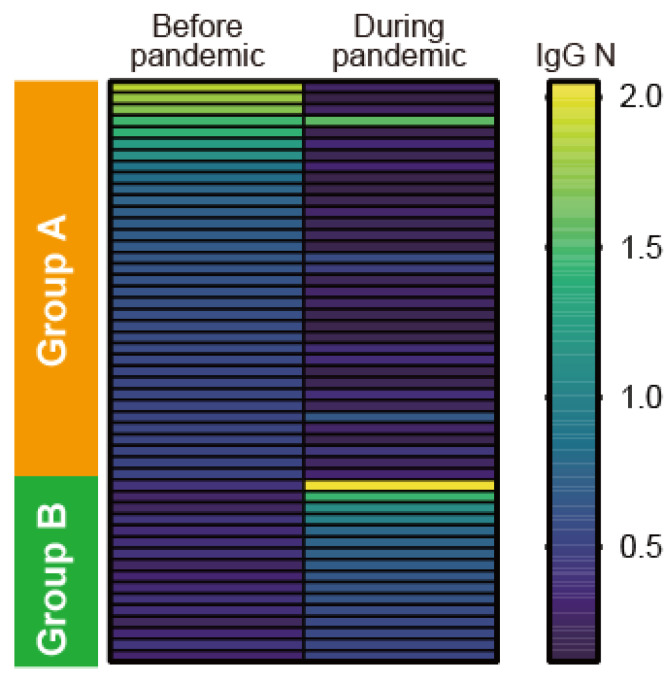
Heatmap images of IgG NC titers of serial serum samples from the same cases obtained before and during the COVID-19 pandemic (*n* = 219). One hundred sixty-eight serum samples (76.7% of tested cases) had no IgG N before and in 2020 (not depicted here). A total of 32 of 35 patients that were positive for IgG N before 2020 became negative in 2020 (indicated as group A), whereas 16 of 184 patients that were negative for IgG N before 2020 became positive in 2020 (group B). Yellow and blue cells depict high and low IgG NC titers (OD values at 450 nm absorbance), respectively.

The above data indicated that the positive rate of IgG N during the pandemic (6.6%) was relatively lower compared with that before the pandemic (13.3%). Lifestyle dramatically changed during the pandemic and potentially reduced the frequency of hCoVs exposure, which might result in the low frequency of IgG N positivity during the pandemic. Since 219 patients’ sera from 368 pre-pandemic cases were obtained in 2020, we evaluated the serial changes of IgG N titers before and during the pandemic in these 219 cases. We found that 32 of 35 IgG N positive patients before the pandemic showed negative IgG responses to N during the pandemic, whereas 16 of 184 IgG N-negative patients had positive IgG responses to N during the pandemic (Figure 5). These data suggested that IgG N titers are variable and not stable, potentially associated with the status of being exposed to pathogens, which might be related to lifestyle changes such as wearing masks and adhering to social distance during the COVID-19 pandemic in 2020. 

### 3.3. Humoral Immunity and Clinical Outcome in Critical COVID-19 Patients

**Figure 6:**
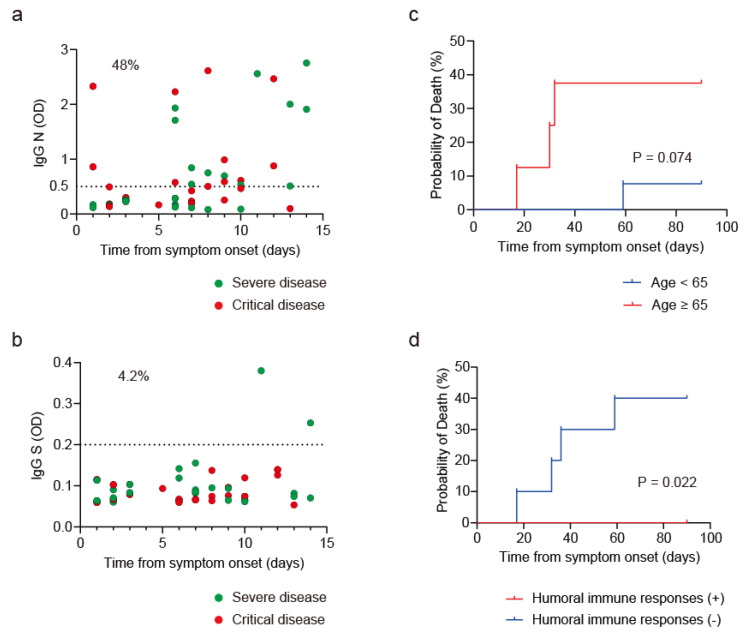
Humoral Immunity to SARS-CoV-2 in hospitalized COVID-19 patients. (**a**) IgG N titers of COVID-19 patients diagnosed with severe (green circles) or critical (red circles) disease. (**b**) IgG S titers of COVID-19 patients diagnosed with severe (green circles) or critical (red circles) disease. (**c**) Kaplan–Meier survival curves of critical COVID-19 patients according to age (blue bar; age < 65, red bar; age ≥ 65). (**d**) Kaplan–Meier survival curves of critical COVID-19 patients with (red bar) or without (blue bar) humoral immune responses to SARS-CoV-2 N.

We evaluated the value of IgG N measurements on the clinical outcome of COVID-19 patients. IgG responses to N and S were evaluated in 48 COVID-19 patients who received intensive care; 27 and 21 of these patients were diagnosed with severe or critical disease on admission, respectively. IgG N elevation was already present in 23 patients (48%), whereas only 2 patients (4.2%) showed IgG S elevation within 14 days (Figure 6a,b). Rapid elevation of IgG N was detected in a subset of COVID-19 patients within 7 days, whereas IgG S elevation was only noted at 11 days or later from symptom onset (Figure 6b). These data clearly indicated the different immune responses to N and S in severe/critical COVID-19 patients. Rapid IgG N elevation could be attributed to the boost effect due to the presence of humoral immune memory acquired by prior hCoVs infection, whereas the lack of rapid IgG S elevation was due to this being the participant’s first exposure to the unique S protein of SARS-CoV-2. Since none of severe COVID-19 patients died, we focused on the characterization of the clinical outcome of 21 critical disease patients. Among the known risk factors such as age, obesity, hypertension and diabetes, age was associated with high mortality with borderline significance (Figure 6c, *p* = 0.074). Moreover, all 11 patients with humoral immune memory to N survived, whereas 4 of 10 without humoral immune memory died (Figure 6d, *p* = 0.022), suggesting a role of humoral immunity in reducing mortality.

## 4. Discussion

Recent evidence suggests the pre-existence of humoral and cellular immunity cross-reacts with SARS-CoV-2 proteins potentially acquired by seasonal endemic hCoVs infection before the pandemic [3,4,5,6]. Although its role on the outcome of COVID-19 remains elusive, a recent study indicated that T cell immune responses may have played a fundamental role in the clinical outcome of COVID-19 patients who recovered or died during intensive care hospitalization [7]. In general, children are less susceptible to severe COVID-19, and a recent study suggested that a significant proportion of children had detectable cross-reactive antibodies to SARS-CoV-2 proteins potentially evoked by endemic hCoVs infection [2]. Indeed, B cells in human tonsillar tissues obtained from children who were negative for COVID-19 prior to the pandemic could generate SARS-CoV-2 reacting antibodies [8]. These data suggest the role of pre-existing humoral and cellular immunity in cross-reacting to SARS-CoV-2 in terms of disease severity and clinical outcome for COVID-19 patients. 

A recent study indicated that pre-existing polymerase-specific T cells expansion plays a crucial role in the clearance of SARS-CoV-2 before the clinical manifestation of COVID-19 [9], suggesting that replication complex proteins as well as spike proteins could be targets for vaccines against SARS-CoV-2 infection. Consistently, our data indicated that cross-reactive humoral immunity to SARS-CoV-2 N might have preventive effects on the mortality of critical COVID-19 patients. Indeed, a study suggested that recent endemic hCoVs infection was associated with less-severe COVID-19 [10]. SARS-CoV-2 N might enhance the infectivity of S particles [11], which may be abolished by the humoral immune responses targeting N. Thus, measurements of humoral immunity potentially acquired by endemic hCoVs may have a prognostic value for the clinical outcome of COVID-19 patients.

Our data demonstrated that about 50% of critical COVID-19 patients in Japan showed antibody responses to SARS-CoV-2 N. A recent study utilized a single cell sequencing approach to clarify the evolution of memory B cells acquired by SARS-CoV-2 [12]. Interestingly, with severe COVID-19, substantial populations of endemic hCoVs-reactive antibody-secreting cells were identified, signifying preexisting immunity evoked by SARS-CoV-2. Importantly, although monoclonal antibodies targeting SARS-CoV-2 N were rapidly generated, they showed non-neutralizing and non-protective effects on SARS-CoV-2 infection using an animal model. Consistently, our data showed that humoral immune memory to SARS-CoV-2 N did not prevent infection, indicating the importance of vaccinations specifically targeting SARS-CoV-2 S RBD.

Our study has several limitations. A primary limitation of this study was that the number of severe/critical COVID-19 patients recruited was small, and we could not fully compare the protective effects of humoral immune status and other known risk factors such as age, obesity, hypertension and diabetes by multivariate analysis due to the small sample size. This study only evaluated the IgG reactivity to SARS-CoV-2 N and S but no other structural/non-structural proteins. Furthermore, serum samples were collected only once at different timepoints after symptom onset on the day of hospitalization, due to the shortage of medical resources and staff, which hampered the time-course analysis of IgG N and S in these patients. Besides, we arbitrarily hypothesized that the presence of IgG N within 10 days after symptom onset in COVID-19 patients might be due to the presence of immune memory to SARS-CoV-2 N acquired by prior hCoVs infection. However, multiple factors can affect both the induction and duration of antibody responses. Immune memory should be ideally evaluated using a B cell ELISPOT assay using N antigen, which we could not conduct due to technical issues at this time. Disease severity is the strongest risk factor for COVID-19 patients’ mortality irrespective of IgG N responses. Future studies are urgently required to test the frequency of memory B cells responding to SARS-CoV-2 N in the general population.

## 5. Conclusions

Subjects had humoral immunity to SARS-CoV-2 N before the pandemic, and early humoral immune responses were observed in approximately 50% of hospitalized severe/critical COVID-19 patients in Japan. Although this humoral immunity did not prevent infection, it might prevent critically ill patients from dying.

## Data Availability

Data are available from the corresponding author upon reasonable request.
